# Tinea InVersicolor: A Rare Distribution of a Common Eruption

**DOI:** 10.7759/cureus.6689

**Published:** 2020-01-17

**Authors:** Matthew Ferry, Lydia Shedlofsky, Andrew Newman, Yebabe Mengesha, Brooke Blumetti

**Affiliations:** 1 Dermatology, Ohio University Heritage College of Osteopathic Medicine, Athens, USA; 2 Dermatology, Affiliated Dermatology, Scottsdale, USA

**Keywords:** dermatology, tinea versicolor, pityriasis versicolor, malasseiza, mycosis, inverse

## Abstract

Tinea versicolor (TV), or pityriasis versicolor, is one of the most commonly occurring superficial mycoses. Typically, this condition is characterized by fine scaly hyper or hypopigmented macules and patches distributed on the trunk and upper extremities. Diagnosis is often based on clinical presentation. A Wood’s lamp examination or potassium hydroxide (KOH) preparation test is performed for confirmation. To date, numerous morphologic variants of this condition have been described. Here we present an inverse papular variant that, to our knowledge, has only been previously reported once. This case represents another unique presentation of TV and serves to highlight the clinical variety of this common mycosis.

## Introduction

*Malassezia* yeasts have been known to cause dermatologic disease for over 150 years [1]. While it is part of the normal human skin flora, this genus has been pathologically implicated in several skin conditions. One such condition is tinea versicolor (TV) [2]. TV, or pityriasis versicolor, is one of the most commonly occurring superficial mycoses. While it has a worldwide distribution, the highest incidence can be found in tropical climates where rates can be as high as 50% [3]. Generally, TV presents with characteristic fine scaly hyper or hypopigmented macules and patches located on the trunk, neck, and upper extremities. The diagnosis often entails a clinical assessment and confirmation via skin scraping with potassium hydroxide (KOH) preparation [4]. While most cases of TV follow a classic pattern, numerous varieties of TV have been described. In recent years, atrophic, papular, imbricate, and folliculocentric forms of TV have been reported [2,5-8]. This report depicts a rare presentation of this common disease, which the authors have termed “Tinea InVersicolor”.

## Case presentation

**Figure 1:**
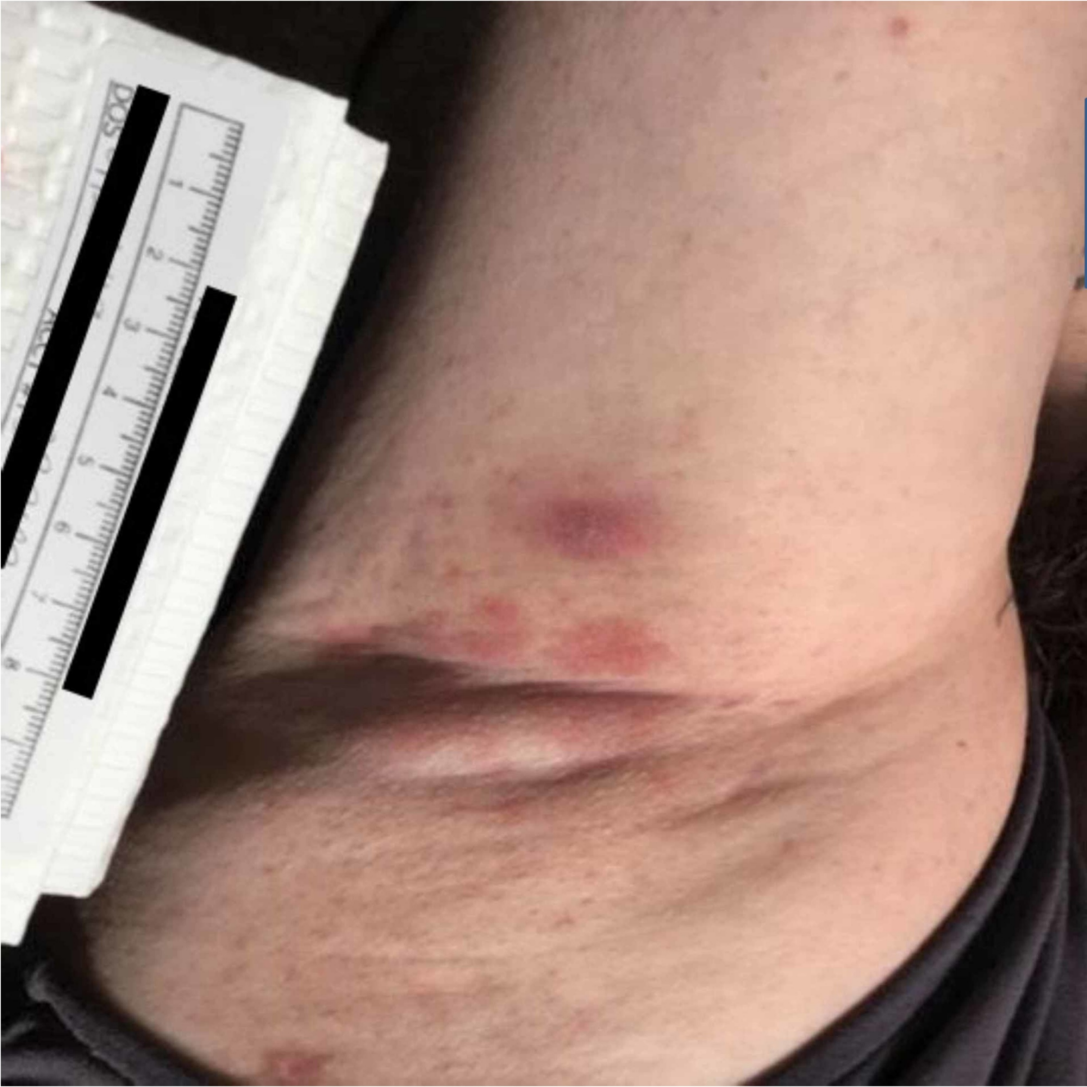
Patient’s right axilla demonstrates a well-demarcated erythematous and papular rash with minimal scale

**Figure 2:**
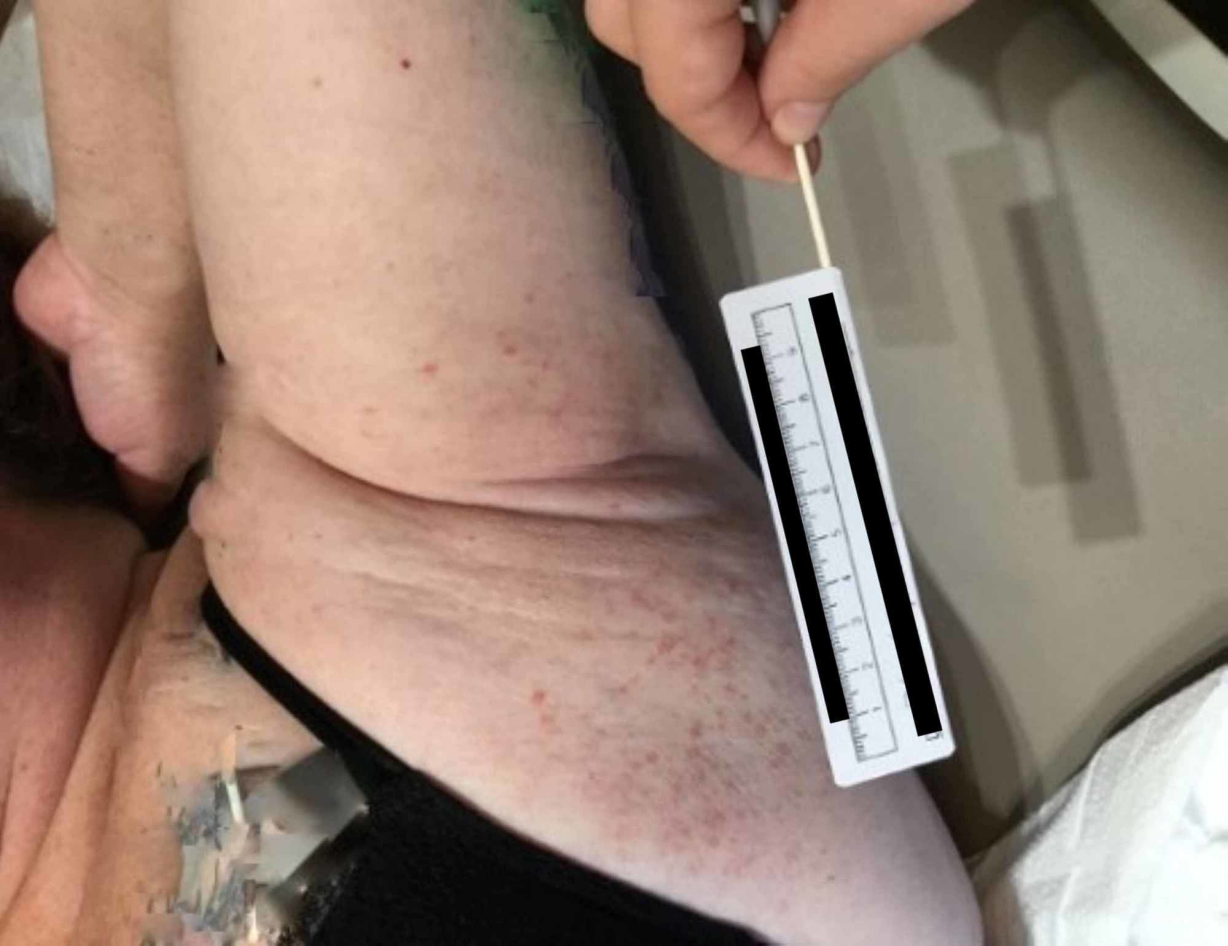
Patient’s left axilla demonstrates the papular eruption extending from her axillary vault to her inframammary tissue (patient’s tattoos have been digitally altered to maintain anonymity)

A 47-year-old Caucasian female with psoriasis presented to an Arizona outpatient dermatology clinic with concerns of a rash in the bilateral underarms with extension to the underside of both breasts. The patient described her condition as red, bumpy, and associated with an occasional burning sensation and pruritus. Her symptoms had persisted for three months. She was initially treated with a short burst of clobetasol ointment and ciclopirox 0.77% gel. The patient reported worsening of the rash with ciclopirox and partial improvement with clobetasol. While the patient denied the use of new skin-care products, medications, or recent travel prior to her rash onset, she did admit to the use of “crystal deodorant”, coconut oil, and hemp oil. Physical exam revealed a well-demarcated, sporadically configured, and bright red papular rash with minimal scale located in the bilateral axilla, which extended to the bilateral inframammary skin (Figures 1 and 2). The rest of the physical exam including examination of the hair and nails was unremarkable.

Based on history and visual morphology, a Wood’s lamp examination and shave biopsy were performed. Upon Wood’s Lamp examination, the involved areas appeared to fluoresce a red-orange color. Shave biopsy revealed stubby hyphae and round spores within the cornified layer, consistent with the diagnosis of TV. 

The patient was placed on fluconazole 300 mg orally every week for four weeks. At a follow-up visit three weeks after starting fluconazole, the patient reported a drastic improvement in her condition. Notably, the patient reported that pruritus had decreased and the rash had begun to clear within 7-10 days of the first administration of the oral anti-fungal medication.

## Discussion

The building of the differential diagnosis for this case started with a consideration of the papulosquamous reaction pattern described by Grooper in 2001 [9]. Given the erythematous, mildly scaly papules defining our patient’s rash, conditions such as infectious intertrigo (i.e. erythrasma, candidiasis, tinea), psoriasis, contact dermatitis, inverse pityriasis rosea, Darier disease, and Grover disease were considered. Due to patient body habitus and intertriginous distribution, an infective intertrigo was initially favored [10]. A lack of response to ciclopirox gel and mild improvement with clobetasol ointment supported a bacterial etiology, such as erythrasma, over candidiasis or dermatophytosis. A review of the patient’s skin-care regimen revealed the use of numerous products including several natural remedies.

In their 2018 review of contact allergens, Uter et al. discuss the rise in contact dermatoses associated with both cosmetic and natural skin-care products [11]. Such evidence, in addition to the observable rash morphology, supports both allergic and irritant contact dermatoses as plausible differentials. While our patient did reveal a history of psoriasis, her psoriasis was not known to present with this morphology or inverse pattern, making this diagnosis less likely. Though infectious intertrigo and contact dermatitis were highest on our differential, neither Darier nor Grover disease could be ruled out by history and physical exam alone. Darier disease, a keratinocyte adhesion disorder, commonly presents on the chest within intertriginous areas, and Grover’s disease presents in a similar pattern with associated pruritus [12,13]. To further distinguish our top differentials, clinical and histological tests were performed.

The patient had initially been referred from an allergist for consultation after her skin-prick testing had revealed environmental allergens severe enough to qualify for immunotherapy. Subsequently, the patient had undergone patch testing with 80 allergens, which had been negative. Her history revealed that the timeline lent credence to a more chronic condition, and so inverse pityriasis rosea was dropped to a lower position on the list of differentials. During the initial office visit, a Wood’s lamp examination and shave biopsy were performed to distinguish among the differentials. The Wood’s lamp, first described in 1903, is a source of ultraviolet light in which all other wavelengths of the visible spectrum are filtered out [14]. False-positive and negative results with the Wood’s lamp, attributed to external factors including skin-care products, have been described in the literature [15]. Our patient's investigation included the Wood’s lamp, which was used to identify a possible superficial skin infection such as erythrasma or TV. These conditions normally fluoresce coral red and yellow/orange respectively under the lamp. With the exam room lights off during the test, the involved areas of our patient’s rash appeared to illuminate red-orange. Additionally, a shave biopsy was performed, which revealed stubby hyphae and round spores within the cornified layer, consistent with the diagnosis of TV.

*Malassezia*, a genus of fungi known previously as *Pitryosporum*, comprises 14 species that are predominately lipophilic, and several of these species are known to be pathogenic [16]. *Malassezia globosa* is the most common etiology of TV [17]. While these eukaryotic organisms generally serve a commensal role on human skin, rapid proliferation and transformation to their hyphal form are pathognomonic for the lesions of TV. While the exact reasons for pathogenic transformation remain unknown, several environmental and biologically inherent influences have been reported to serve as risk factors for the disease. From an environmental perspective, the prevalence of TV in tropical regions approaches 50% [3]. Sunlight and the associated increase in pilosebaceous secretions have been shown to support *Malassezia* proliferation and likely account for the robust prevalence in tropical areas [17]. According to Metin et al., other important risk factors for the disease include the use of oil-containing skin-care products and the application of corticosteroids [2]. Generally, these substances provide a substrate for yeast metabolism and create a nurturing microenvironment for yeast overgrowth [2,17]. In hindsight, our patient exhibited several risk factors for disease. The warm Arizona climate, use of multiple skin-care products, and initial treatment with topical corticosteroids might have all contributed to the development and persistent nature of TV in our patient. In considering the elicited history, the reported improvement with topical steroids directed clinical thinking away from a mycotic etiology. The lack of improvement with topical ciclopirox is likely secondary to a lack of compliance with treatment. The reported improvement with topical steroids is a clinical outlier, rooted in the reduction of *Malassezi*a-induced inflammatory cytokines [17]. This case should encourage physicians to consider TV as a differential in situations where topical steroids have elicited a partial treatment response. 

Most commonly, TV lesions present as hyperpigmented or hypopigmented macules and patches distributed on the trunk and upper extremities [2,4,5,17,18]. Generally, these lesions exhibit a superficial scale and lack both clinical and histologic signs of inflammation. Atypical variants of TV have been previously reported [2,5-8]. To date, variations of both clinical distribution and morphology have been described. In their discussion of anatomic distributions of TV, Varada et al. state that the trunk and upper extremities may be involved in up to 95% of the cases. The most common non-classic distributions include the scalp and groin. Interestingly enough, Varada et al. [6] also report that the flexural creases, namely the axilla and inguinal folds, are commonly spared in cases of widespread infection. Our patient's anatomic involvement was limited to the intertriginous areas of the trunk, including bilateral axilla and inferior breast tissue. To our knowledge, only two cases of TV presenting with a predominantly inverse anatomic distribution have been reported [7]. Rudolph et al. describe two cases of TV with an inverse presentation. The first case involves a 32-year-old male with TV distributed solely in the bilateral axilla and inguinal folds, while the second case depicts a 23-year-old pregnant female with TV distributed solely in her perineal region and superior thighs [7]. In line with the findings of Rudolph et al., the authors herein support the idea of expanding the differential of inversely distributed eruptions to include TV.

In addition to the unusual anatomic distribution, our patient exhibited an unusual TV morphology: well-defined and sporadically configured papules with minimal scale. In recent years, several morphologic variants of TV have been described. These include atrophic, papular, imbricate, and folliculocentric forms [2,5-8]. In their discussion of a papular variant, Pouldar et al. describe brown flat-topped papules coalescing into reticulated plaques in a 26-year-old female [8]. The lesions seen in our patient remained as discrete papules, were erythematous in appearance, and may represent a newly reported form of the condition. Given the inimitable morphology and extensive list of differential diagnoses, shave biopsy, rather than potassium hydroxide (KOH) preparation, was performed. It is likely that a KOH preparation would have likely expedited the diagnosis. In fact, all the cases of abnormal TV reviewed for this publication had a positive KOH preparation [5-8]. Furthermore, in their discussion of bedside dermatologic tests, Wanat et al. report the sensitivity and specificity of KOH preparation to be 88% and 95% respectively for the diagnosis of TV [19]. Moving forward, the authors of this article advise clinicians to keep the anatomic and morphologic variants of TV in mind and to consider KOH preparation to speed up the diagnosis of this easily treatable condition. 

## Conclusions

In summary, this case showcases an unusual inverse presentation of the commonly occurring superficial mycosis, TV. To our knowledge, TV displaying a similar type of papular eruption with a predominantly intertriginous distribution has been reported only once before. The authors favor labeling this TV variant as “Tinea InVersicolor” because it is simple yet descriptive. Our discussion of “Tinea InVersicolor” will hopefully serve as a useful reminder of the variability often seen with common skin diseases. Finally, we hope our findings will encourage clinicians to lower their threshold for including TV in the differential diagnosis of an inverse rash suspected to be erythrasma or another infectious intertrigo, contact dermatitis of the underarms, or a truncal papular eruption like Darier disease or Grover disease.
